# Collaborative improvement in Scottish GP clusters after the Quality and Outcomes Framework: a qualitative study

**DOI:** 10.3399/BJGP.2020.1101

**Published:** 2021-07-27

**Authors:** Huayi Huang, Emily R Jefferson, Mark Gotink, Carol Sinclair, Stewart W Mercer, Bruce Guthrie

**Affiliations:** Usher Institute, University of Edinburgh, Edinburgh.; Division of Population Health and Genomics, School of Medicine, University of Dundee, Dundee.; Usher Institute, University of Edinburgh, Edinburgh.; Public Health Scotland, Edinburgh.; Usher Institute, University of Edinburgh, Edinburgh.; Usher Institute, University of Edinburgh, Edinburgh.

**Keywords:** data science, leadership, primary health care, qualitative research, quality improvement

## Abstract

**Background:**

Scotland abolished the Quality and Outcomes Framework (QOF) in April 2016, before implementing a new Scottish GP contract in April 2018. Since 2016, groups of practices (GP clusters) have been incentivised to meet regularly to plan and organise quality improvement (QI) as part of this new direction in primary care policy.

**Aim:**

To understand the organisation and perceived impact of GP clusters, including how they use quantitative data for improvement.

**Design and setting:**

Thematic analysis of semi-structured interviews with key stakeholders (*n* = 17) and observations of GP cluster meetings (*n* = 6) in two clusters.

**Method:**

This analytical strategy was combined with a purposive (variation) sampling approach to the sources of data, to try to identify commonalities across diverse stakeholder experiences of working in or on the idea of GP clusters. Variation was sought particularly in terms of stakeholders’ level of involvement in improvement initiatives, and in their disciplinary affiliations.

**Results:**

There was uncertainty as to whether GP clusters should focus on activities generated internally or externally by the wider healthcare system (for example, from Scottish Health Boards), although the two observed clusters generally generated their own ideas and issues. Clusters operated with variable administrative/managerial and data support, and variable baseline leadership experience and QI skills. Qualitative approaches formed the focus of collaborative learning in cluster meetings, through sharing and discussion of member practices’ own understandings and experiences. Less evidence was observed of data analytics being championed in these meetings, partly because of barriers to accessing the analytics data and existing data quality.

**Conclusion:**

Cluster development would benefit from more consistent training and support for cluster leads in small-group facilitation, leadership, and QI expertise, and data analytics access and capacity. While GP clusters are up and running, their impact is likely to be limited without further investment in developing capacity in these areas.

## INTRODUCTION

Quality improvement (QI) is increasingly recognised as being integral to clinical practice.^1^^,^^2^ In the UK, national initiatives to improve quality have included both externally imposed pay-for-performance in the Quality and Outcomes Framework (QOF), alongside professionally led activity such as the Royal College of General Practitioners’ network of regional QI champions,^3^ all of which operate in the current context of high demand, workforce shortages, and limited resources.^4^

GP clusters were created in Scotland when QOF was abolished in 2016. These are geographical groupings of practices, intended to deliver locality-based collaborative QI. Since 2017/2018, all Scottish GP practices have been members of one of 147 clusters of differing sizes depending on local circumstances and geography. A cluster typically includes four to eight practices with 20 000–40 000 registered patients,^5^ regularly meeting to identify quality issues important in the locality and working collaboratively to meet the healthcare needs of their population (see Supplementary Appendix S1 for details).

GP clusters therefore represent a significant change from the top-down pay-for-performance of QOF to a locality-based approach to improving quality, similar to ‘quality circles’, which have a long history in European primary care.^6^^,^^7^ The idea of clusters is consistent with previous and current Scottish health policy highlighting the value of professionalism,^8^ in contrast with the more market-oriented, central, target-setting policies in England^9^ where QOF continues (although in scaled-down form). But important commonalities exist across the current wave of primary care reform across the four nations of the UK.^10^ This includes an emphasis on better use of existing quantitative data and data analytics for primary care improvement^4^^,^^11^ (reflecting a prevalent wider assumption in the value of this in UK policy communities); however, it is unclear whether and how GP clusters access or use data for improvement.

This qualitative study aimed to examine current improvement work and use of data in Scottish GP clusters, with a particular interest in whether and how quantitative data (for example, from platforms such as the Scottish Primary Care Information dashboards and Care Information Gateway)^12^^,^^13^ was used for improvement.

## METHOD

### Overall design

Qualitative analysis was undertaken of interview and observational data collected from professionals (referred to as ‘improvers’ throughout) involved in primary care improvement and/or GP clusters.

### Sampling and recruitment

GP cluster professionals and other primary care improvers were purposively sampled to ensure heterogeneity in their work roles, in relation to level of involvement in improvement (national, regional, or local) and disciplinary perspective. Cluster Quality Leads (CQLs [GPs convening cluster meetings]), Practice Quality Leads (PQLs [GPs from member practices attending cluster meetings]), practice managers, and health board managers/representatives were approached, alongside NHS LIST (Local Intelligence Support Team) analysts, who are the main data analytics professionals currently involved from Public Health Scotland. Meetings of two clusters in one health board were also observed between November 2019 and September 2020 (with a gap during the first COVID-19 lockdowns), sampled to be clusters perceived by health board managers to vary in their maturity.

**Table:** How this fits in

The Quality and Outcomes Framework (QOF) dominated UK primary care quality improvement until recently, but, as it has been reduced in scale (abolished in Scotland), interest is growing in other ways to deliver improvements. This article shows that, in the post-QOF landscape, clusters are trying to improve the quality of health care across their member practices; they are working primarily to an internally driven agenda, with limited access to national or local quantitative data on quality of care. While GP clusters are up and running, their impact is likely to be limited without further investment in cluster development and capacity, particularly in relation to leadership, quality improvement expertise, and data analytics access and capacity.

Informed consent was taken at the time of interview, with participant information sheets shared beforehand. For observations, written informed consent was taken from all cluster members before observations started, and reaffirmed at subsequent meetings.

### Data collection

Interviews were one-to-one, in-depth, and semi-structured. They were conducted with national and health board level improvers, as well as cluster members working in the South East regions/localities. They were a mix of face-to-face and telephone interviews, complemented by non-participant observations of cluster meetings of the two participating clusters (pseudonymised as ‘Corbett’ and ‘Munro’). The interviews explored participant perceptions of GP cluster work, and the use of data in quality improvement in the cluster and more widely. Interviews were complemented by the cluster meeting observations to better understand possible barriers and facilitators to data-driven improvement. The semi-structured data collection was iteratively refined in light of emerging findings on these topics.^14^^,^^15^ Interviews were transcribed by GDPR-compliant transcribers, complementing contemporaneous field notes made for the interviews and cluster meetings. Data collection started in late 2019 but was suspended from March 2020 to July 2020 owing to COVID-19 (which meant that fewer observations and interviews were completed than originally planned). NVivo 12 Pro was used to manage data organisation and analysis.

### Data analysis

Analysis drew on the interplay between the authors’ a priori ideas, and those emerging from the participants and field observations.^16^^,^^17^ In seeking to inform policy and practice decision making, a domain summary-based approach to thematic analysis was undertaken (organised under the three domains later on in the results), but focused on capturing the ‘essence of meaning’ to the research team within the analytic narrative told for each of these major domains of interest.^18^^,^^19^ Drawing on participants’ views of their improvement and data usage experiences in broad terms (and research observations), data interpretation occurred through the following:

- data familiarisation;
- generation of codes (that is, the initial ‘units of meaning’ developed by qualitative analysts);^20^
- searching for candidate themes in light of mutually compatible units of meaning from the data;^20^^,^^21^
- reviewing these candidate themes; and
- defining/redefining, and naming the final ‘meaning units’ and themes presented as results.

The first author carried out all data collection, coding, and analysis, with other co-authors also reading a selection of transcripts and/or contributing to the qualitative data analysis below.

## RESULTS

Seventeen primary care improvers participated in one-to-one interviews (lasting 30–145 min), of which seven were members of the two sampled GP clusters (Table 1). All interviewees had other professional roles alongside their primary care improvement roles. Six cluster meetings were observed (ranging between 40–90 min in length), with >20 cluster members observed overall.

**Table 1.** Participant characteristics

**Participant**	**Sex**	**Working on clusters at a national/ regional/local level**	**Role**	**Member of one of the selected clusters**
P1	F	Local/regional	Clinical services support manager supporting multiple clusters within their HSCP	Yes
P2	F	Local	Principal LIST information analyst	No
P3	F	Local	Cluster Quality Lead	Yes
P4	M	National	Retired GP active in policy and improvement	No
P5	M	National	Academic GP (practising 1 day/week)	No
P6	F	National	NHS Health Scotland officer, Primary Care Information dashboard working group member	No
P7	M	National	Principal analyst within a national healthcare statistics division	No
P8	F	Local	Practice manager	Yes
P9^a^	M	National	GP involved in national work to develop clusters	No
P11	F	Local	Practice manager/practice business partner	Yes
P12^a^	F	National/local	GP and Cluster Quality Lead involved in national work to develop clusters	No
P13	F	Local	Cluster Quality Lead	Yes
P15^a^	M	National	GP involved in national work to develop clusters	No
P16	F	Local/regional	Senior LIST information analyst	No
P17	M	Local	Practice Quality Lead	Yes
P21	F	National	NHS National Services Scotland information analyst	No
P22	F	Local	Practice manager	Yes

a *Recruited because they also talked with colleagues in Scottish GP clusters (on behalf of the Royal College of General Practitioners), to understand how they felt about life after the Quality and Outcomes Framework. Covering all of Scotland between them, these three participants shared their reflections, which were based on observing and discussing cluster development with* >*100 Scottish colleagues (including* >*70 Cluster Quality Leads and* >*10 Practice Quality Leads). HSCP = Health and Social Care Partnership. LIST = Local Intelligence Support Team.*

### Views from national/regional improvers in primary care

From a national perspective, the coming together of individual practices into clusters was likened to the bringing together of *‘five or six little ships* […] *each starting with its own distinct practice culture and sometimes with “a very strong ethos of us and them”’* (participant [P]5, academic GP). CQLs were perceived to be central in enabling practices to work together for some, but in context of uncertainty about what their cluster should focus on:

*‘So you give* [secondary care data] *to GPs, but in a vacuum of not really knowing what they’re supposed to be doing. And feeling quite potentially exposed. Because we [CQLs] don’t know what this cluster is* […] *There’s already an anxiety that at some point somebody’s going to come along and say right, “You’ve got to do quality assurance.” So you’re going to have to mark your peers and the staff in the clusters. And start kind of telling your peers that they’re not doing well enough.’*

(P12, CQL involved in national work)

Developing a shared vision was seen as crucially dependent on the prior leadership and quality improvement experiences of their CQLs/PQLs:

*‘I think there are some clusters that straight from the very beginning were very proactive* […] *GPs involved in a lot of leadership at local or national level or done a lot of quality improvement work before, they seem to almost thrive in the cluster environment, knowing how to make changes, people to approach, how to go about things* […] *Whereas some other clusters that had people who didn’t really have that experience* […] *were the ones that struggled initially* […] *still have a large amount of variety even today.’*

(P15, GP involved in national work)

In buying into the vision of the GP cluster as a mechanism for data-driven primary care reform/improvement, national improvers perceived variations in cluster level engagement with the analytic resources and tools offered:

*‘So, I guess this is where it’s probably still quite early days* [for the clusters and data] *. I think clusters and data are very related but they maybe don’t know it yet, maybe the clusters don’t know how important they are in this space yet.’*

(P6, NHS Health Scotland officer)

National stakeholders also described variability in the support provided to GP clusters, with the potential to significantly affect their improvement activity.

First, clusters receive varying amounts of administrative support from their Health Boards or Health and Social Care Partnerships (HSCPs). A CQL involved in national work (P12) observed that some, but not all, clusters had dedicated administrative support from their board (as in one of the two selected clusters observed). A few clusters/boards also had quality improvement advisors working with them. Two of the national improvers (P12 and P5) noted that the clusters that had struggled to find an effective way of working often had minimal access to administrative support and external improvement expertise.

Second, the LIST data analytic support received varied between clusters and health boards:

*‘Some GPs just haven’t heard of them* [LIST analysts] *. So in some places, it seems like LIST analysts have spent all their time with the HSCPs, who have their own agenda, and their own stuff to do* […] *big thing with HSCP is health and social care integration. So some GPs I’ve spoken to haven’t even heard of LIST analysts.’*

(P5, academic GP)

On the other hand, for a senior LIST analyst looking after around eight clusters in a large territorial health board, the data work they do with the clusters seemed relatively straightforward:

*‘I think just because the relationships built* [are] *quite strong, so they don’t feel like it’s too much hassle to ask us for data to help with any data, any projects.’*

(P16, senior LIST information analyst)

In summary, interviews with improvers at the national/regional levels suggest considerable uncertainty from colleagues post-QOF, regarding whether clusters should focus primarily on activities generated internally or externally by the wider healthcare system. Variations in leadership and improvement experiences were also seen as crucial factors in developing a shared vision for each cluster, alongside variable buy-in, administrative, and analytics support in the process of implementing data analytics for GP clusters (as observed by the participants).

### Observation of cluster meetings

The cluster meetings observed had agendas with both standing/reoccurring items and emerging issues usually raised by the CQL. In both clusters, meeting attendees came prepared to work together to solve problems identified and discussed by group members (both before and after COVID-19). But there were differences between the two clusters in the social dynamics of the group.

The social relationship between the CQL and other meeting members in the Munro cluster, appeared to be free-flowing and close, with the conversation often ranging beyond topics strictly related to work, and peppered with jokes in good humour. One of the CQLs came across as more experienced in small group leadership and improvement. But the other cluster’s CQL shared (in one-to-one interview) that they had taken the CQL role because nobody else had wanted to do it. This was their first wider leadership role, and they more commonly engaged in coaxing contributions from group members compared with the first CQL (mentioned above).

Despite these differences in meeting dynamics, both clusters actively encouraged shared learning and development of local solutions to problems members identified as being important to them. Other than the move to an online method of meeting, the basic running/shape of all the cluster meetings was broadly similar, both before and after the onset of the first COVID-19 lockdowns in March 2020.

In cluster meetings observed before COVID-19, both CQLs consistently strove to champion an improvement agenda, for example, through leading discussion on the standing agenda item of learning from significant events. CQLs were often the first to share learning from their recent experiences in areas both more and less clearly related to improvement. Other examples of improvement activity included cluster members discussing issues such as the need to collectively absorb additional patients from the impending retirement of a single-handed GP (late 2019 meeting) and in sharing thoughts around further developing a mechanism to reduce the amount of patient-related correspondence GPs automatically receive that does not require GP input or response before being archived (early 2020). For example, this could include screening normal results or certain types of hospital discharge letters.

However, there was less evidence of the data analytics policy aspiration being championed in these meetings. Quantitative data and quantitative ideas were mentioned only briefly; one example being a discussion in the Corbett cluster attended by nonmember visitors, focused on how to address the cluster’s status as an outlier in anticholinergic prescribing. These external attendees shared their experiences of their own prescribing quality improvement project (in anticholinergic prescribing), with the cluster ending up agreeing to further explore the potential of doing something similar. In the same meeting, the idea of a single point of access for palliative care referrals was presented by another pair of visitors to the group as a possible improvement project for the cluster.

During COVID-19, cluster members valued cluster meetings for sharing learning and managing rapidly changing ways of working. For instance, in a Corbett cluster meeting, sharing of recent experiences of significantly increased phone bills occurred, with discussion of the use of internet-based alternatives to traditional landline phones. In a Munro cluster meeting, members raised and discussed a shared concern around practices’ stock control of personal protective equipment (PPE), and whether employees contracted to but ‘external’ to a practice could make use of the practice stock. Group members shared their rules of thumb for this decision with each other (in light of ambiguous policy guidance), concluding that a consistent policy for their cluster would be to offer PPE from the practice’s stock only when the ‘external’ affiliated professional was not a shared resource across multiple surgeries, but affiliated only to their surgery.

From these observations, evidence can be seen of internally driven shared learning in these clusters, focused on local problems important to member practices in serving their clinical populations. Some differences were observed between the two clusters, in their social dynamics and existing relationships between group members. Data analytics/quantitative ideas were occasionally used to support the learning shared and observed in these meetings, but there was far more conversation around topics relating to improving the sustainability and quality of service provision. The CQLs often led these group discussions, sometimes letting the group dynamics take over in working through an agenda relevant to the day-to-day opportunities and challenges of their group.

### Using data for improvement

In observation, communication and evidence-sharing during the cluster meetings was largely qualitative, in that members usually described personal or practice experiences and plans, rather than referring to quantitative data on quality. However, members did express interest in quantitative data in their interviews. The interview data provided further evidence for the top-down view on QOF, in suggesting that national/regional/local performance indicators were indeed defined *for* Scottish practices previously, as part of QOF and other extrinsically incentivised improvement schemes. Cluster members reflected on the lack of such indicators or definitions of ‘good quality’ in the new contract, leaving some uncertain as to how to compare the quality of their practice work with others. For example, one practice manager suggested that some practices still used QOF indicators for their benchmarking because they had performed well on these before.

In their interviews, both CQLs raised access to data as a significant barrier. Data security was perceived as overly complex and a major barrier to accessing nationally provided data tools:

*‘So I can access data for my practice, I can’t compare my practice against next door’s practice, unless that data is available centrally and relatively easily accessible* […] *flaming hoops and walk across hot coals to actually get access* […] *I’ve lost my log-in and I’m just not wanting to go through it again. It’s not terribly accessible.’* (P13, CQL)

One CQL suggested that a simpler single point of access integrating improvement data with QI ideas, methods, and practical resources might also help:

*‘If somehow I could just have one single sign-on that would get me to all the data collection websites that were out there. Actually one website that’s got everything pulled together because at the minute there’s Scottish Therapeutics Utility, SPIRE, etc. So one website focused on quality improvement that’s got the data there as well.’* (P3, CQL)

As well as better access to data resources where cluster members could pull down data, a support manager working across multiple clusters suggested that enabling managers to also access existing data (or at least timely regular standard reports pushed out by LIST) would enable better integration of the available data analytics with existing cluster work/meeting cycles.

Cluster members were interested in quantitative comparisons across practices and clusters, but interviewees had concerns about data quality and coding. They were interested in comparisons between practices *within a cluster*, comparisons between clusters *within a health board/ HSCP*, and in how a group of practices or clusters are performing in comparison with the rest of Scotland.

When pushed for clarification, one CQL said that data aggregated at cluster level would help their work most, because of the concern that focusing on outliers *within* a cluster might risk intra-cluster conflict from some members who might feel vulnerable and attacked, whereas a whole cluster being an outlier could instead spur shared action. However, other cluster members said they could work with comparisons between practices in the cluster, if the quantitative data coming in was perceived to correctly reflect their own local circumstances.

In further discussing the topic, one GP (P9) shared a recurring theme in their conversations with Scottish colleagues around life after QOF. This was that primary care is seen to have evolved from a past position of general disengagement with the need to improve data quality to support large-scale quantitative analysis, to a position of practices now becoming willing to do what they can to improve data quality by improving their clinical coding. Technological variation between the two main Scottish clinical IT systems of EMIS and Vision were seen (elsewhere in the interviews) as significant in allowing practices to fine-tune available clinical code sets to variation in specific local needs. Historical variability in practice-level QOF-coding and Read codes training approaches were also perceived by participants as drivers of current coding practices (Read codes are a thesaurus of clinical terms widely used for coding of medical events, conditions, diagnoses, and so on).

## DISCUSSION

### Summary

Since the abolition of QOF, some Scottish GP clusters face uncertainties in achieving a balance between internally and externally driven QI work. The two observed clusters had both developed a primarily internally driven approach to their collaborative improvement, although both also invited external visitors to propose ideas for future improvement projects. The ‘qualitative approach’ to communication and evidence sharing mentioned in the results refers to the sharing of non-numerical sources of information^22^ as observed in these cluster meetings. Observations from the field evidenced a natural user preference towards sharing and elaborating on tacit elements from experiential/professional knowledge (difficult to comprehensively document/fully codify);^23^ alongside limited use of explicit elements from *‘hard* [quantified] *data’* (P4) sources and platforms in these meetings.

Key barriers identified by national and board improvers to further progress in a collaborative improvement included the variable administrative/managerial and data support, and leadership experience available to clusters. This was reflected in the way that the two clusters observed worked, in providing both a forum for shared learning and collaborative work facilitated through words and conversation. At a cluster level, participants in the larger project of collaborative improvement also generally remain interested in the idea of quantitative analytics. Key barriers to integrating and using such data in existing processes included difficulties in accessing data and concerns about data quality.

Both CQLs consistently strove to champion and facilitate the improvement agenda in their meetings, providing further evidence of ‘normalisation’^24^ and integration of quality improvement ideas, principles, and methods into contemporary clinical practice. In contrast, the data analytics aspiration of Scottish policy seemed to be at a comparatively earlier stage of development (both before and during COVID-19). Meanwhile, this study’s observations show that both clusters worked together as internally driven, informal networks of local learning and peer support to address the diverse problems and opportunities from their everyday work. Encouragingly, this informal network persisted even in the face of the recent pandemic, with members using the cluster to support each other in the face of rapidly changing needs. A key message from the interviews was that the use of data for collaborative improvement needs to take place with care, so as to benefit rather than harm the social dynamics of these clusters and the needs of their members.

### Strengths and limitations

A strength of the current study is its use of both interview and observational data, and its iterative and adaptive approach to learning about the lived experiences of improvement work in Scottish GP clusters.^25^ Limitations to ‘reader generalisability’^26^^,^^27^ include the fact that the cluster meetings of only two GP clusters were observed, although these were purposively sampled to be at different levels of maturity in developing as productive social groups.^6^^,^^7^ In addition, data were not collected from the practices themselves, since the focus was on the clusters, but future work to understand how cluster work translates into practice-based improvement will be important. Finally, COVID-19 disrupted the latter parts of the study recruitment, meaning not all of the ≥30 study participants originally intended were recruited, but it was interesting to observe that the GP cluster model did appear to help practices share learning in response to COVID-19-driven rapid change.

### Comparison with existing literature

Similar to findings from a study involving Welsh cluster leads,^28^ some participants were also uncertain about how clusters will evolve. Uncertainty for these Welsh leads related to future funding arrangements for their clusters, but the participants in this study were concerned more with tensions around expectation for CQLs to ‘police’ or collaboratively engage with their cluster members. This tension could relate to cluster members’ sense of loyalty towards their practices, in avoiding ‘change for change’s sake’.^29^ The internally driven collaboration observed in this study is arguably a firm foundation on which clusters can improve their integration with local public health and social care agencies, structures, and processes.^28^^,^^30^

A nationwide survey of Scottish GPs in the second half of 2018^31^ found that participants believed that GP clusters were, on average, ‘up and running’, but needed more support to improve quality of care. The current study shows that administrative and data analytics support for clusters is perceived to vary widely between clusters, as are the prior leadership and quality improvement experiences of CQLs/PQLs, implying a need for more training. The two clusters observed were clearly ‘up and running’ in the sense of providing an internally driven, informal network of learning and peer support for members (both before and during COVID-19). But the maturity of the two clusters seemed a little different, in relation to both the prior leadership experiences of the two CQLs and the varying history of prior collaboration between members.

### Implications for research and practice

Better training and support for CQLs is likely to help drive the agenda for collaborative improvement in Scottish GP clusters forward. This will be particularly relevant in developing CQLs’ leadership, QI expertise, and access to/use of data analytics, perhaps in integration with training and development offerings and processes from local health boards. Such training likely needs to become a part of medical undergraduate and postgraduate education,^32^ if collaborative working between practices is to become the ‘new normal’. In context of the high service demand and limited workforce and delivery capacity,^4^ additional administrative and managerial support would undoubtedly help to support the delivery of service improvement via clusters.

While the redefinition or reconceptualisation of complex interventions (for example, data analytics for primary care and GP clusters) is normal for professional and lay participants in health care,^33^ it is useful to pause to reflect on where we are up to in both the expected and unexpected outcomes of the current interventions,^34^^–^36 in light of a Scottish improvement landscape in flux. Measures for improvement were still regarded as key by some participants in the recent Scottish primary care reforms. But cluster members’ persisting interest in practice level through to national comparisons are currently stymied by a complicated and burdensome situation. These issues of data access may in part explain the focus on more qualitative ways of sharing knowledge and learning observed (drawing more on what was previously tacit rather than explicit sources), in the context of existing drivers like safety checklists, practice culture/professional ethos, and health system infrastructure. While LIST analysts are starting to be seen as ‘one of us’ by some clusters, the extent to which this is occurring seems inconsistent across the country.

While practices are ‘up and running’ in clusters across the country, there seems to be emerging tensions within Scotland. On the one hand, the study’s data show evidence of *localised* frameworks of quality improvement developing at a cluster level,^11^ being created through these emerging inter-practice collaborations, and the observed iterative approach to small-scale change, learning, and adaptation.^37^ But pushing data analytics into these clusters risks negative effects on small-group dynamics, relationships, and future appetite for collaboration, through formal comparisons between members.

Further research questions might seek to understand the following:

- What is the proper balance between internally versus externally generated ideas, measures, and activities for improvement?
- What is the acceptable balance of quantitative and qualitative data^22^ in light of these user preferences^38^ for more qualitative ways of sharing knowledge and learning (from a user-centred technology research perspective)?
- How can the added value from data analytics be harnessed in a setting where tensions emerge from the sociopolitical dynamics of local versus national governance of improvement and health care more broadly?
- In light of the sort of collaborative improvement and learning observed, how can data analytics be harnessed to enrich the learning and group dynamics of an emerging organisational unit (the GP cluster), to encompass both sources of everyday success and failure in maintaining safety?^39^

In context of the Scottish model, this should help transform quality improvement from driven mainly by its ‘clinical elites’, into the wider policy vision for data-enabled grassroots change, while avoiding reinventing the QOF under another name.
